# Supplementary material for: Mechanistic insights into dual-active liver and blood-stage antiplasmodials
Source: mBio. 2025 Nov 24;17(1):e02423-25. doi: 10.1128/mbio.02423-25 (PMC12802172; doi:10.1128/mbio.02423-25)
Supplement: Supplemental Figures — Figures S1-S3. [file mbio.02423-25-s0001.pdf]

Supplementary Information: Mechanistic Insights into Dual-Active Liver and Blood-Stage Antiplasmodials

Supplementary Figure 1: Growth recovery after drug washout.

Supplementary Figure 2: TCMDC-141334 and TCMDC-140674 target *P. falciparum* cGMP-dependent protein kinase (PfPKG).

Supplementary Figure 3: Parasite egress phenotype of PKG inhibitors

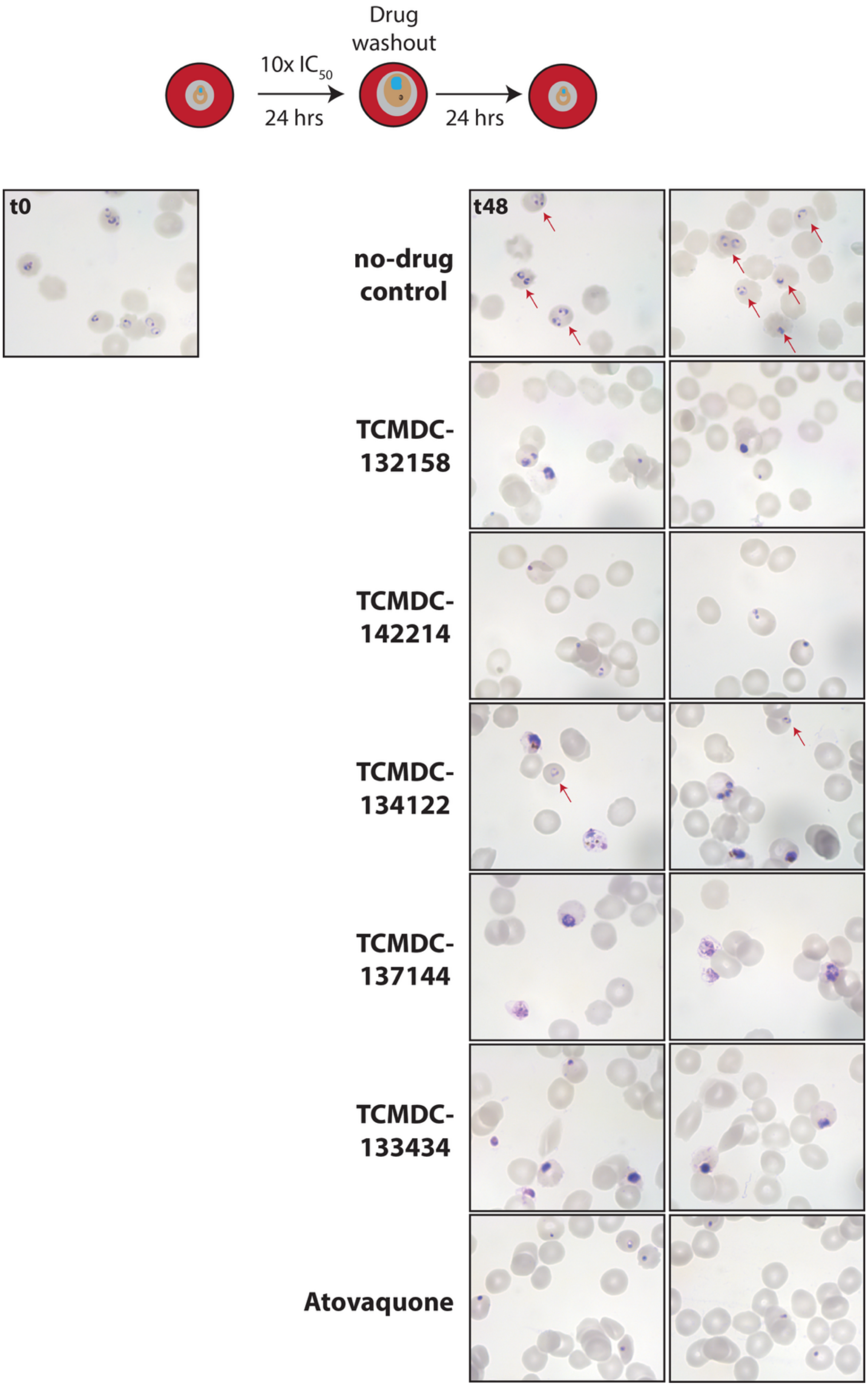

Supplementary Figure 1: **Growth recovery after drug washout.** Ring-stage parasites were treated with 10× IC_{50} concentrations of the indicated compounds for 24 hours, followed by drug washout. Parasite reinvasion (rings indicated by red arrows) was then assessed after 24 hours using Giemsa staining. Atovaquone was included as a control. Continued growth inhibition post-washout was observed for all tested compounds, with the exception of TCMDC-134122 for which a small number of rings were observed.

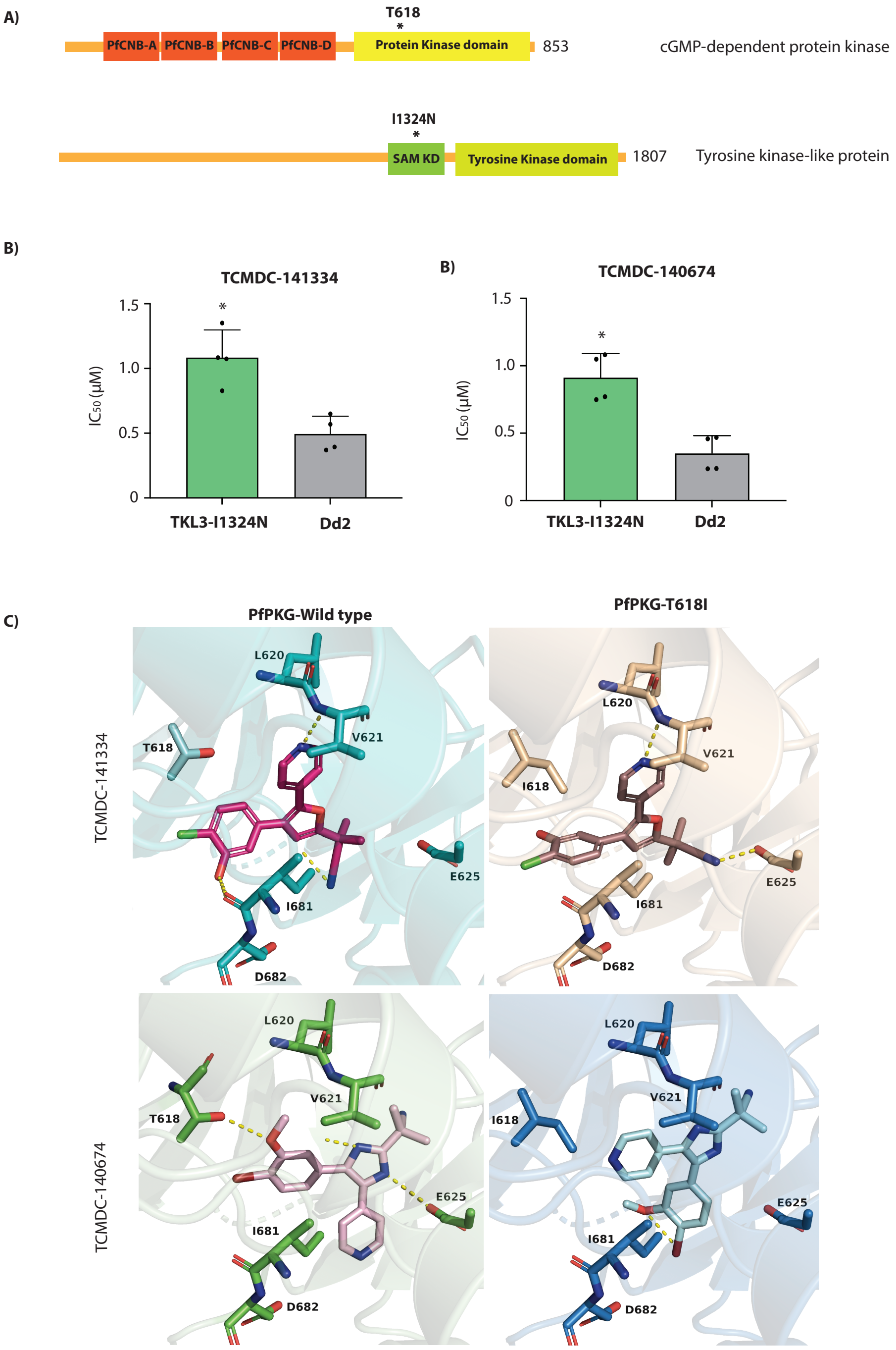

Supplementary Figure 2: TCMDC-141334 and TCMDC-140674 target *P. falciparum* cGMP-dependent protein kinase (PfPKG). (A) Schematic representation of the domain architecture of PfPKG and PfTKL3. **(B)** CRISPR-edited parasite lines carrying the *Pf*TKL3 I1342N mutation exhibited reduced sensitivity to both TCMDC-141334 and TCMDC-140674. The Dd2 parental line was included as a reference. Each dot represents a biological replicate (n = 4); bars indicate mean ± SD and statistical significance determined by Mann-Whitney *U* tests (*p<0.05, **p<0.01). **(C)** Molecular docking models showing the binding of TCMDC-141334 and TCMDC-140674 to both wild-type PfPKG and the PfPKG-T618I mutant.

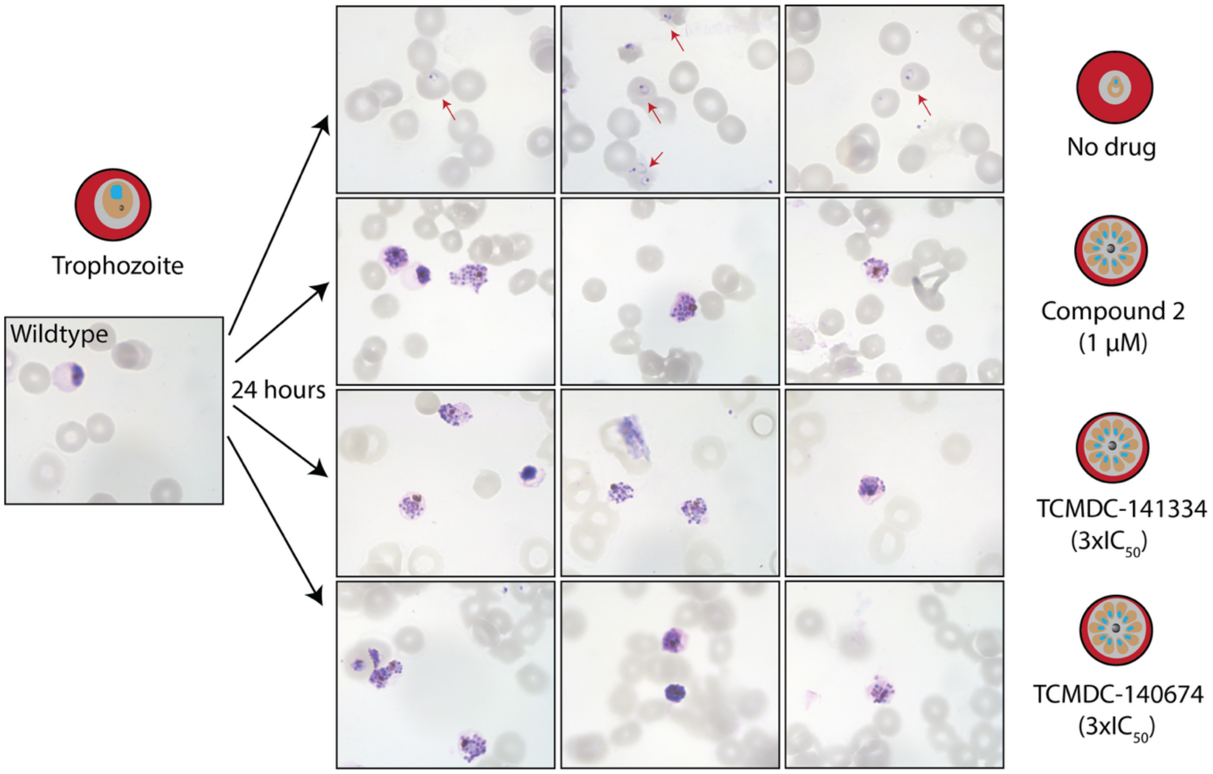

Supplementary Figure 3: Parasite egress phenotype of PKG inhibitors. Tightly synchronised *P. falciparum* 3D7 parasites at the trophozoite stage were treated with 3×IC_{50} concentrations of TCMDC-141334 and TCMDC-140674. Compound 2 (1 μM) was included as a positive control. Giemsa staining showed that, in the untreated culture, parasites progressed to the ring stage after 24 hours (red arrows), indicating normal egress and invasion. In contrast, parasites treated with TCMDC-141334, TCMDC-140674, or Compound 2 exhibited a similar phenotype, consistent with egress inhibition.
